# Mode of action of lipoprotein modification enzymes—Novel antibacterial targets

**DOI:** 10.1111/mmi.14610

**Published:** 2020-10-12

**Authors:** Simon Legood, Ivo G. Boneca, Nienke Buddelmeijer

**Affiliations:** ^1^ Institut Pasteur Unité Biologie et Génétique de la Paroi Bactérienne Paris France; ^2^ CNRS, UMR 2001 « Microbiologie intégrative et Moléculaire » Paris France; ^3^ INSERM Groupe Avenir Paris France; ^4^ Université de Paris Sorbonne Paris Cité Paris France

**Keywords:** diacylglyceryl transferase, in vitro activity assays, inhibitors, lipoprotein, N‐acyl transferase, phospholipid, signal peptidase, X‐ray crystal structure

## Abstract

Lipoproteins are characterized by a fatty acid moiety at their amino‐terminus through which they are anchored into membranes. They fulfill a variety of essential functions in bacterial cells, such as cell wall maintenance, virulence, efflux of toxic elements including antibiotics, and uptake of nutrients. The posttranslational modification process of lipoproteins involves the sequential action of integral membrane enzymes and phospholipids as acyl donors. In recent years, the structures of the lipoprotein modification enzymes have been solved by X‐ray crystallography leading to a greater insight into their function and the molecular mechanism of the reactions. The catalytic domains of the enzymes are exposed to the periplasm or external milieu and are readily accessible to small molecules. Since the lipoprotein modification pathway is essential in proteobacteria, it is a potential target for the development of novel antibiotics. In this review, we discuss recent literature on the structural characterization of the enzymes, and the in vitro activity assays compatible with high‐throughput screening for inhibitors, with perspectives on the development of new antimicrobial agents.

## Graphical abstract

The function, structure and antibiotic target potential of the lipoprotein modification enzymes in bacteria is discussed in this review. Upon translocation in the membrane by the Tat or Sec translocon, lipoproteins are post‐translationally modified by the sequential action of Lgt, Lsp and Lnt. The pathway is essential in proteobacteria, and is important for virulence in actinobacteria. Novel alternative modification enzymes are also described.

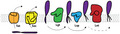

## 1 INTRODUCTION

**FIGURE 1:**
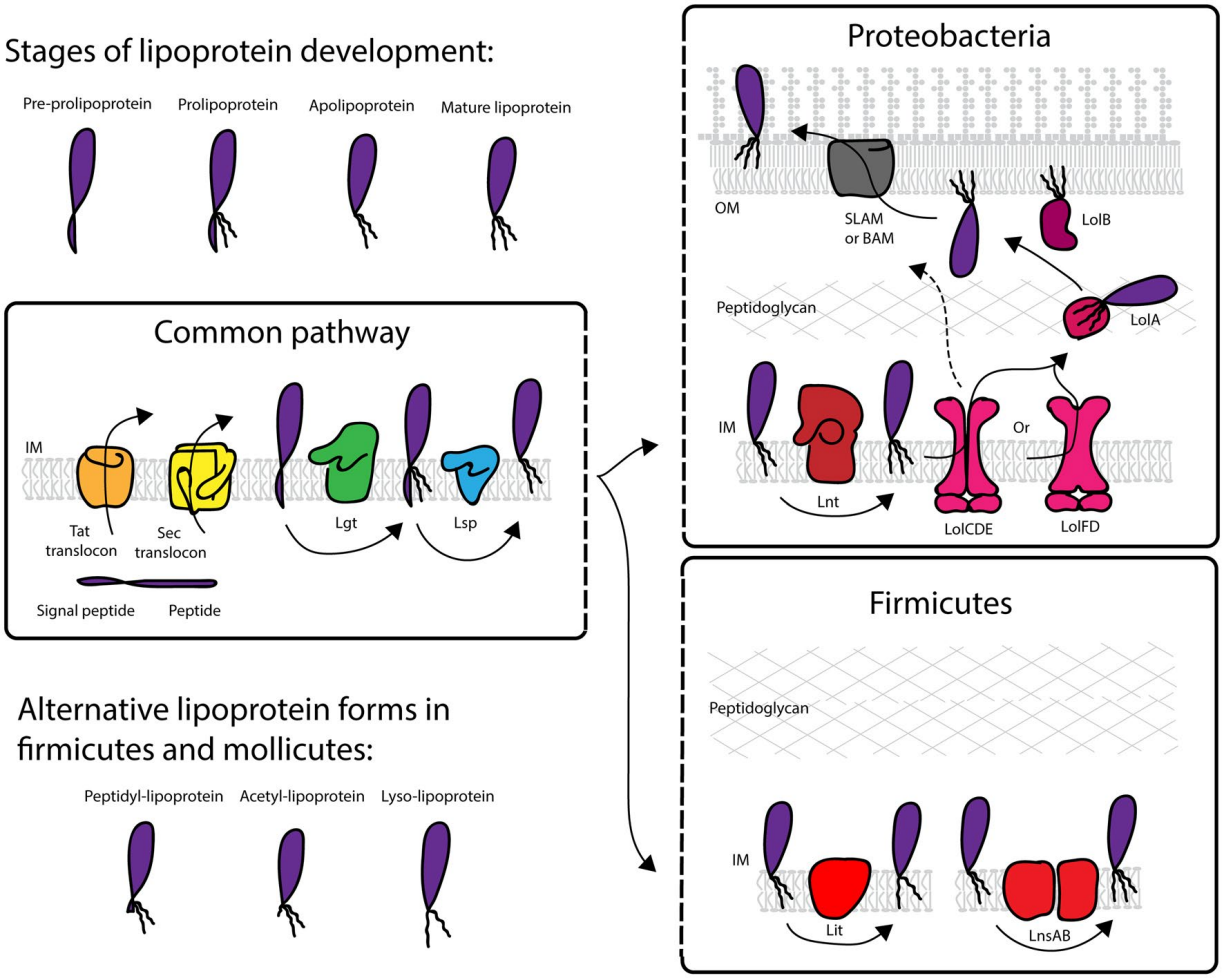
The lipoprotein biosynthesis pathway. Pre‐prolipoprotein is translocated across the cytoplasmic membrane via the Sec or Tat translocons and the signal peptide is embedded in the membrane with the functional part exposed to the extra‐cytoplasmic space (external to the cell in monoderm bacteria, the periplasm in diderm bacteria). The lipobox region of the signal peptide is recognized by Lgt that transfers diacylglyceryl from phosphatidylglycerol to an invariable cysteine in the lipobox forming prolipoprotein. The prolipoprotein is recognized by signal peptidase Lsp, which cleaves the signal peptide below the diacylated cysteine to form apolipoprotein. In proteobacteria, Lnt then N‐acylates the apolipoprotein by transferring an acyl group from phosphatidylethanolamine to the α‐amine group of the terminal cysteine to form a mature lipoprotein. The LolCDE (or LolFD) ABC‐transporter transfers the lipoprotein to a periplasmic chaperone, LolA, which escorts the lipoprotein to the outer membrane where LolB inserts the triacylated protein into the membrane. In some monoderm bacteria, alternative forms of lipoproteins have been identified, including peptidyl‐lipoprotein, acetyl‐lipoprotein, and lyso‐lipoprotein. In firmicutes, Lit forms lyso‐lipoprotein from apolipoprotein and LnsA and LnsB are both involved in N‐acylation of apolipoprotein resulting in triacylated lipoprotein

Volkmar Braun first discovered bacterial lipoproteins in 1973 through the identification of a fatty‐acid modification of Lpp, or Braun's lipoprotein, in *E. coli* (Hantke and Braun, 1973). Through early biochemical and genetics studies and more recent structural analysis, the lipoprotein modification pathway is increasingly well understood. A general consensus exists regarding the well‐studied tripartite stages of the lipoprotein modification pathway. Upon insertion into the cytoplasmic membrane, a diacylglyceryl group is added to the lipoprotein, the membrane‐spanning signal peptide is cleaved and the protein stays membrane anchored by its diacylglyceryl moiety. Finally, N‐acylation results in the formation of mature triacylated lipoprotein (Figure 1). In diderm bacteria, including proteobacteria and some high GC content Gram‐positive bacteria, including *Streptomyces*, *Corynebacteria*, and *Mycobacteria*, lipoproteins are triacylated following this classical pathway, although in some instances Lnt and/or Lsp are not essential components for cell viability (discussed below). In monoderm bacteria it was long thought that only diacylated lipoproteins existed; however, recent studies illustrate that alternative lipid modifications occur in firmicutes and mollicutes, but not all enzymes catalyzing these reactions have been identified (Armbruster and Meredith, 2017; Asanuma et al., 2011; Kurokawa et al., 2009; Navarre et al., 1996) (Figure 1). An intra‐molecular N‐acyltransferase (Lit), which generates a lyso‐form lipoprotein, is one such enzyme that has been characterized (Armbruster et al., 2020; Armbruster and Meredith, 2017). A recent study also identified two genes, *lns*A and *lns*B, in *Staphylococcus* species that are involved in N‐acylation of lipoproteins (Gardiner et al., 2020). Lipoproteins are mainly located in the outer membrane and on the cell surface of proteobacteria (Wilson and Bernstein, 2016). The lipoprotein outer membrane localization (Lol) machinery is the canonical pathway for trafficking to the outer membrane, but recent studies suggest alternative Lol‐independent mechanisms and other transport systems may exist in parallel.

The roles of lipoproteins in cellular processes are numerous, and include cell wall biogenesis, efflux of harmful substances and virulence. They also signal the innate immune system through recognition by Toll‐like receptors where the lipid moiety is essential (Kovacs‐Simon et al., 2011; Nguyen and Gotz, 2016). The essential nature of the pathway in proteobacteria is likely due to the essential function of some lipoproteins in outer membrane physiology, such as LptE in LPS translocation (Wu et al., 2006) or BamD in outer membrane protein assembly (Malinverni et al., 2006; Misra et al., 2015; Onufryk et al., 2005). In *Mycobacteria*, lipoprotein LpqW plays a key role in cell wall biogenesis and has been hypothesized as the reason for Lgt essentiality (Rainczuk et al., 2012; Tschumi et al., 2012).

New and exciting insights have been obtained in recent years on the molecular mechanism of lipoprotein modification enzymes and their structural arrangements in the membrane. The increase in antimicrobial resistance demands the identification of novel targets for the development of novel antibiotics. Due to its essential nature in proteobacteria, the accessibility of the catalytic domains of the enzymes, and the existence of high‐throughput in vitro assays, the lipoprotein modification pathway is a promising target.

## 2 ENTERING THE PATHWAY

Lipoproteins are synthesized in the cytoplasm as pre‐prolipoproteins and contain an N‐terminal signal sequence harboring a critical recognition sequence known as the lipobox. The signal sequence has a positively charged n‐region, a hydrophobic h‐region, and a lipobox containing c‐region (Babu et al., 2006; Hayashi and Wu, 1990; von Heijne, 1989). The lipobox takes a standard form of [LV]^−3^ [ASTVI]^−2^ [GAS]^−1^ [C]^+1^, based on lipoprotein sequences from multiple organisms, where the invariant cysteine is the site of lipid modification that becomes the first amino acid in the mature lipoprotein (Babu et al., 2006). Variations in lipobox sequences have been reported but the invariant cysteine residue is always present (Valente et al., 2007).

The pre‐prolipoprotein is translocated into the cytoplasmic membrane via the Sec translocon (Hayashi and Wu, 1985; Kosic et al., 1993; Watanabe et al., 1988) or Tat translocon (Shruthi et al., 2010a; 2010b; Thompson et al., 2010). The posttranslational targeting of secretory proteins by SecB and co‐translational targeting of inner membrane proteins by signal recognition particle (SRP) converge at the Sec translocon (Tsirigotaki et al., 2017). Although the membrane translocation of lipoproteins has not been extensively studied, two examples of lipoproteins in *E. coli*, including Lpp, depend on SRP and YidC for targeting to the Sec machinery (Fröderberg et al., 2004). This may also be the pathway used in spirochetes since they lack SecB and there is no evidence for a functional Tat pathway (Zuckert, 2014). As a result of translocation, the Cys^+1^ in the lipobox is located at the membrane–periplasm interface of the outer leaflet of the cytoplasmic membrane, ready to be modified through fatty acid acylation, with the mature part of the protein located in the periplasm (Figure 1).

## 3 BECOMING MATURE

### 3.1 Diacylglyceryl transfer by Lgt

**FIGURE 2:**
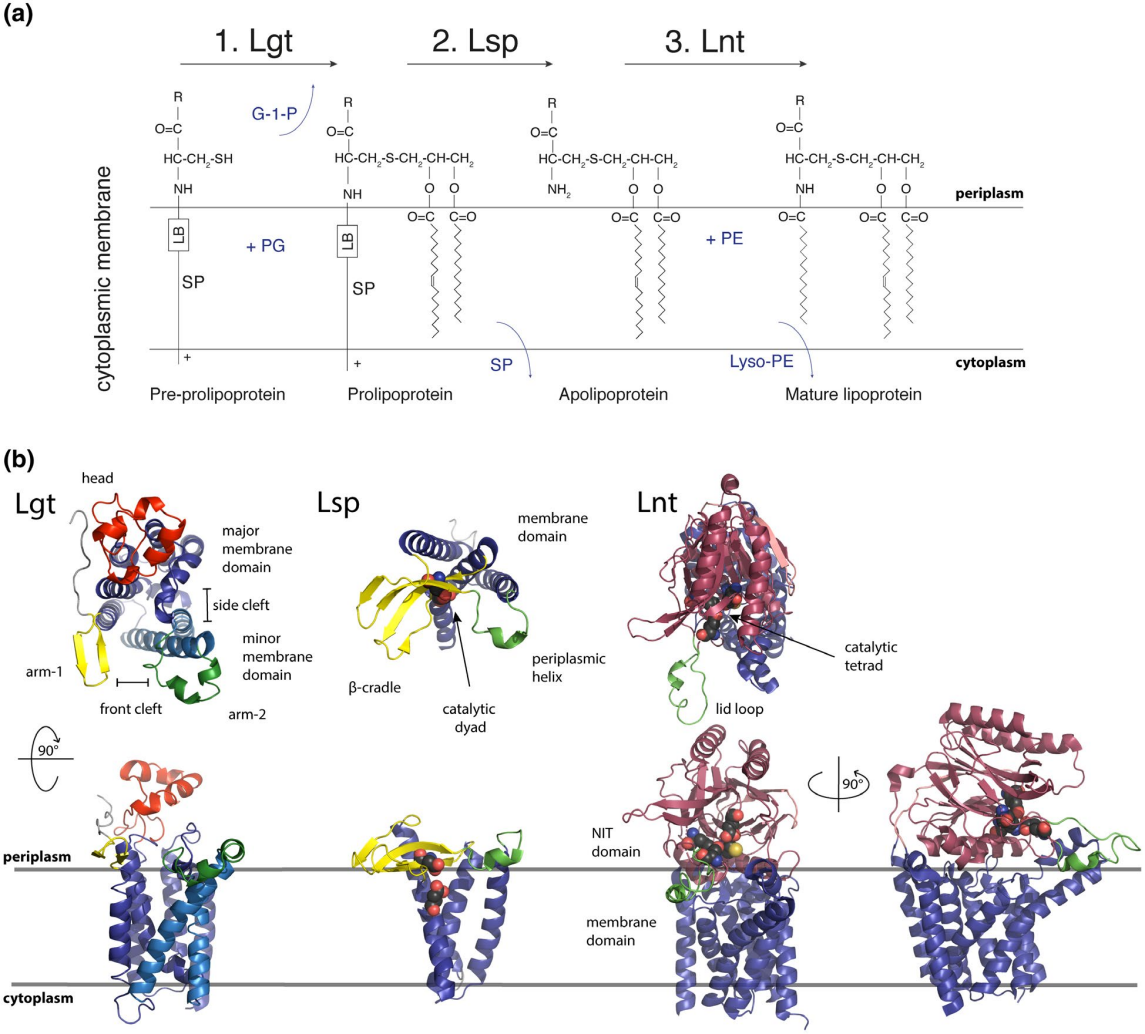
(a) Chemical modifications of the stages of lipoprotein biosynthesis. (b) X‐ray crystal structure of Lgt, Lsp, and Lnt from proteobacteria (respective PDB files: 5AZC, 5DIR, 5N6H). Lgt is composed of a periplasm exposed head domain, two arm domains that rest on the periplasmic leaflet of the cytoplasmic membrane, and a minor and major body domain. Front and side clefts are formed between the two body domains where substrate is likely to enter (front cleft) and exit (side cleft). The arms possibly provide guidance and stabilizing functions for the incoming pre‐prolipoprotein and the reaction is believed to occur within the central cavity. Lsp is composed of three domains, a β‐cradle and periplasmic helix which both protrude from the membrane domain and rest on the periplasmic face of the cytoplasmic membrane between which the substrate binds. The two critical, catalytic residues which act to cleave the signal peptide are found in the upper half of the membrane domain (spheres). Lnt has two distinct domains: a NIT (nitrilase) domain where N‐acylation occurs and a membrane domain. A flexible lid‐loop (green) protrudes from the enzyme and may be correlated to binding and accessibility of substrate. The catalytic triad (spheres) is observed inside the NIT domain close to the lid‐loop. G‐1‐P, glycerol‐1‐phosphate; LB, lipobox; PE, phosphatidylethanolamine; PG, phosphatidylglycerol; SP, signal peptide

In the first modification step, phosphatidylglycerol: prolipoprotein diacylglyceryl transferase (Lgt) recognizes the lipobox Cys^+1^ of the bilayer embedded signal peptide, and transfers a diacylglyceryl group from phosphatidylglycerol to the pre‐prolipoprotein, resulting in thioether‐linked S‐diacylglyceryl lipoprotein (Gan et al., 1993) (Figure 2a). Lgt possesses seven transmembrane helices (TH) with the N‐terminus facing the periplasm and the C‐terminus located in the cytoplasm (Daley et al., 2005; Mao et al., 2016; Pailler et al., 2012). The enzyme folds into five domains, a large transmembrane body domain with its seven THs, which in turn consists of a minor (TH2, TH3) and major (TH1, TH4‐7) domain, a head domain extending into the periplasm, and two arms (arm‐1, arm‐2) in the periplasm facing away from the head domain parallel to the membrane (Figure 2b). Arm‐1 is a short β‐hairpin extending from TH1, and arm‐2 consists of two α‐helices extending from the minor body domain. This minor body domain is thought to be flexible and may have a role in guiding the pre‐prolipoprotein substrate into the catalytic center (Mao et al., 2016). A front cleft is formed between the TH1 of the major domain and TH2 of the minor domain, and is a proposed entry site for the two Lgt substrates, pre‐prolipoprotein and phosphatidylglycerol (Singh et al., 2019). A side cleft is formed between TH3 of the minor domain and TH7 of the major domain that functions as the exit portal of diacylglyceryl‐modified product (Figure 2b).

A central cavity in the body domain, whose base is hydrophobic and contains a positively charged region above, with a large opening to the periplasmic side, houses the conserved H103‐G‐G‐L sequence, the Lgt signature motif (G142–G154) and other essential residues as found in *E. coli* (Mao et al., 2016; Pailler et al., 2012). Within the cavity are two phosphatidylglycerol binding sites. At the first binding site, near the front cleft, arm‐2 and Y26 interact with the phosphate group of the phospholipid. The second binding site is near essential residues R143 and R239 and is thought to be where diacylglyceryl transfer occurs. In the structure, diacylglycerol (DAG) is observed in a pocket formed by essential residues (Pailler et al., 2012; Sankaran et al., 1997), probably representing an intermediate state since DAG is not a substrate nor product of the Lgt reaction. Both alkyl groups pass through the side cleft (Mao et al., 2016). The following reaction mechanism is proposed based on the structural data. The Lgt signature motif binds the lipobox of pre‐prolipoprotein coming in from the side cleft, such that the cysteine is in close proximity to the C3 ester group of phosphatidylglycerol. Upon lipoprotein binding, the thiol group of the cysteine is converted into a reactive thiyl radical via proton release to H103 that, in turn, attacks the ester bond in phosphatidylglycerol, transferring the diacylglyceryl group to the cysteine in the lipobox, releasing glycerol‐1‐phosphate (G1P) through a periplasmic exit.

Several models have been proposed for substrate entry and product exit: (a) the phospholipid substrate occupies the two binding sites simultaneously, and upon catalysis, phospholipid moves from site‐1 to site‐2 for a new round of catalysis and the product exits via the side cleft (Mao et al., 2016); or (b), binding of lipoprotein induces a conformational change that leads to entry of phospholipid in the catalytic site‐2 (Mao et al., 2016). Alternatively, (c) phosphatidylglycerol and the pre‐prolipoprotein both enter through the front cleft into the central cavity, where essential residue R239 acts as a gate that regulates the opening and closing of a loop in the major domain allowing products to leave via the side cleft (Singh et al., 2019).

In the early 1990's a first Lgt in vitro activity assay was reported based on a shift in mobility by high‐resolution gel electrophoresis of a diacylglyceryl peptide, upon incubation with Lgt and phospholipid (Sankaran and Wu, 1994). Crude membrane fractions of bacteria with varying levels of Lgt were used as the enzyme source for the conversion of a synthetic peptide, composed of the first 24 residues of Braun's lipoprotein in the presence of radiolabeled membrane phospholipids (Gan et al., 1993; Sankaran and Wu, 1994). From these studies, the glycerol head group of phospholipid was shown to be specific for Lgt (Sankaran and Wu, 1994). A coupled enzymatic reaction described by (Sundaram et al., 2012) monitors Lgt activity through the formation of G1P, a by‐product of the reaction directly correlated with enzyme activity. Dihydroxyacetone is formed from G1P using a combination of alkaline phosphatase and glycerol dehydrogenase. In a final step, resazurin is reduced to resorufin and fluorescence read‐out monitored as a measure of Lgt activity. Both methods are based on the same *E. coli* strain to overproduce Lgt and the same synthetic peptide substrate, resulting in similar Km values for the peptide. In a recent report, peptide substrate LipoGFP, also containing the N‐terminal sequence of Lpp fused to GFP, was used as Lgt substrate (Mao et al., 2016). This peptide was produced in *E. coli* as a glutathione‐S‐transferase (GST) fusion protein for purification purposes and after cleavage of GST used as substrate. Upon incubation with commercial phospholipids and purified enzyme, formation of diacylglyceryl‐lipoGFP is followed by a shift in migration on SDS‐PAGE and fluorescence detection.

The methods based on gel shift of diacylglyceryl peptides can be used in elaborate kinetic studies on Lgt, however, they are not compatible with high‐throughput screening (HTS) required in the search for and development of novel antibiotics. Even though the resorufin fluorescence‐based assay could be developed for multi‐well plates, the necessity for two additional enzymes requires additional control steps and complicates the HTS set‐up. Other challenges are the chemical nature of the reaction; acylated proteins, phospholipids, and integral membrane enzymes require nonclassical conditions for catalysis, as will be discussed below.

### 3.2 Cleavage of the signal peptide by Lsp

Once diacylation of the lipobox cysteine by Lgt has occurred, Lsp cleaves the signal peptide liberating the α‐amino group of the prolipoprotein (Figure 2a). The X‐ray crystal structure of signal peptidase II (Lsp) from *P. aeruginosa* (Vogeley et al., 2016) and *S. aureus* (Olatunji et al., 2020) reveals two domains; a membrane domain consisting of the four transmembrane helices, with both the N and C terminus located in the cytoplasm (Munoa et al., 1991), and a periplasmic domain consisting of two sub domains—the β‐cradle, resting on the outer leaflet of the inner membrane, and α‐helix, with a single helical turn also resting on the membrane surface (Vogeley et al., 2016). Lsp belongs to the aspartate protease family (Tjalsma et al., 1999), where the catalytic aspartate residues reside at the membrane‐periplasm interface in TH1 and TH4.

The incoming prolipoprotein likely enters between the β‐cradle and the periplasmic helix, which form two arms extending away from the core of the enzyme (Vogeley et al., 2016). The scissile bond between the diacylglyceryl‐modified cysteine and the amino acids at position‐1 in the lipobox extends between the catalytic dyad (D124 and D143 in Lsp of *P. aeruginosa*) (Figure 2b) and is clamped by the β‐cradle and the periplasmic helix (Olatunji et al., 2020; Vogeley et al., 2016). The catalytic site contains a water molecule in a deprotonated state. One aspartic acid residue acts as a base to attract hydrogen from the water molecule and creates a hydroxide that attacks the scissile peptide bond. This generates a tetrahedral intermediate. A second aspartic acid donates a proton to the amino terminus of the peptide, and the tetrahedral intermediate also donates a proton. This causes cleavage of the scissile bond and the substrates dissociate from the enzyme (Paetzel et al., 2002).

Lsp is the only enzyme in the lipoprotein modification pathway with known natural inhibitors. Globomycin is a cyclic peptide produced by *Streptomyces* (Inukai et al., 1978a; 1978b; Nakajima et al., 1978) that shares similarities to the signal peptide of lipoproteins (Inukai et al., 1978b). The second molecule, myxovirescin (also called TA), was isolated from *Myxococcus xanthus* (Rosenberg et al., 1973). The genome of *M. xanthus* encodes four Lsp genes (*lspA1* to *lspA4*) (Konovalova et al., 2010; Paitan et al., 1999; Xiao and Wall, 2014), two of which (*lspA3* and *lspA4*) are located in the myxovirescin biosynthetic gene cluster (Xiao and Wall, 2014). The mechanism of host protection is not fully understood but has been hypothesized due to either (over‐)expression of *lspA3*, which conferred highest resistance when expressed in *E. coli* or regulation in antibiotic levels by LspA4 (Xiao and Wall, 2014). In the *S. aureus* Lsp structures, globomycin and myxovirescin share a 19‐atom core structure bound in the central cavity of the enzyme, blocking the catalytic dyad (Olatunji et al., 2020), and is presumably where the signal peptide of prolipoprotein binds, whereas the macrocycles each occupy opposite sides of the catalytic site.

Proteolytic processing of prolipoprotein by Lsp in vitro was first shown in the early 1980's using a gel shift assay similar to those used in the study of Lgt (Tokunaga et al., 1982; Wu et al., 1983). Prior modification of substrate by Lgt is required for Lsp activity (Tokunaga et al., 1982; 1984). Recent work on the mode of action of globomycin and myxovirescin describe a similar coupled Lgt and Lsp reaction to obtain diacylglyceryl‐modified substrate for Lsp (Olatunji et al., 2020; Vogeley et al., 2016). This study also highlights differences in enzymatic activity and inhibition by globomycin between Lsp enzymes of different bacterial species. Lsp of *P. aeruginosa* is more efficient in processing prolipopeptide than Lsp from *S. aureus* and has a lower inhibitory concentration for globomycin as measured by half maximal inhibitory concentrations (IC50 values) (Olatunji et al., 2020). Slight structural differences are observed between the Lsp enzymes in loop structures involved in keeping the antibiotic in place, and overall surface electrostatic differences between the two enzymes are also likely to play a role. Minimal inhibitory concentrations (MIC) of globomycin on bacterial cell cultures are much higher for *Pseudomonas* and *Staphylococcus* than for *E. coli* (Kiho et al., 2003; 2004). Specific small molecule inhibitors of Lsp were identified in a FRET assay based on processing of a synthetic diacylglyceryl‐lipopeptide containing a fluorophore and quencher (Kitamura et al., 2018). Upon incubation with Lsp, processing of the peptide results in fluorescence of the fluorophore due to loss of the quencher. In an HTS, specific Lsp inhibitors were identified that could be optimized by medicinal chemistry to obtain IC50 values in the nanomolar range (Kitamura et al., 2018).

Lsp is essential for growth in proteobacteria and in *S. coelicolor* (Thompson et al., 2010) and probably also in *S. scabies* since suppressor mutants were readily obtained in attempts to delete *lsp* (Widdick et al., 2011). However, it is not essential in *Corynebacteria* (Dautin et al., 2020) and *Mycobacteria* but an *lsp* mutant in *M. tuberculosis* is attenuated for virulence (Rampini et al., 2008; Sander et al., 2004). The rationale for targeting lipoprotein biogenesis holds true.

### 3.3 N‐acyl transfer by Lnt

Lnt catalyzes a third and final step in the lipoprotein modification pathway, by N‐acylation of the apolipoprotein formed by cleavage of the signal peptide by Lsp. The essential nature of Lnt is not completely conserved in proteobacteria. Recent studies demonstrate that *Francisella tularensis*, *Neisseria gonorrhoeae* (LoVullo et al., 2015), *Neisseria meningitidis* (da Silva et al., 2017), *Acinetobacter* spp (Gwin et al., 2018), and *Helicobacter pylori* (McClain et al., 2020) are viable under laboratory conditions in the absence of Lnt. This phenomenon is possibly related to noncanonical Lol machinery in which LolF functions as LolCE in the translocation of OM lipoproteins, however, the basis and extent of this is not fully understood (see below).

Lnt is a member of the nitrilase superfamily catalyzing hydrolysis or condensation of carbon‐nitrogen and nitrile bonds (Pace and Brenner, 2001). Within the enzyme, a catalytic triad E267, K335, C387 has been proposed for *E. coli* (Vidal‐Ingigliardi et al., 2007). The enzyme exists in a thioester‐acyl intermediate in vivo through acylation of the C387 sulfur group that is blocked for alkylation. Residues E267, K335, and E343 are involved in formation of this stable intermediate (Buddelmeijer and Young, 2010). The X‐ray crystal structure of Lnt was reported by three research groups in quick succession (Lu et al., 2017; Noland et al., 2017; Wiktor et al., 2017) and has been reviewed in greater detail by (Cheng et al., 2018) (Figure 2b). Recently, Wiseman and Hogbom (2020) published a fourth similar structure. Due to the critical role of E343 and its fixed position in all structures, it has been proposed that the catalytic triad is in fact a tetrad (El Arnaout and Soulimane, 2019; Wiktor et al., 2017). Initial proton abstraction from the C387 sulfur by E267 generates a thiolate that in turn attacks the ester linkage between the *sn*‐1 acyl of phosphatidylethanolamine, forming a tetrahedral intermediate stabilized by K335 and an oxyanion hole. When the tetrahedral intermediate collapses, proton abstraction from E267 releases the lyso‐phospholipid by‐product. When the apolipoprotein substrate enters the thioester acyl enzyme, the reaction passes through a second tetrahedral intermediate that forms when the α‐amine of S‐diacylglyceryl‐cysteine in the apolipoprotein attacks the carboxyl carbon between C387 and the acyl chain. The mature lipoprotein is thereby formed and released. The reaction follows a ping‐pong mechanism where lyso‐phospholipid is released before binding of the second apolipoprotein substrate (Hillmann et al., 2011).

The characteristic catalytic domain, as seen in nitrilases, sits on top of the transmembrane domain composed of eight transmembrane helices (Figure 2b). Both termini are in the cytoplasm (Lu et al., 2017; Noland et al., 2017; Robichon et al., 2005; Wiktor et al., 2017; Wiseman and Hogbom, 2020). The nitrilase domain has a characteristic αββα fold and contains a domed cavity with an opening into the membrane domain. A phosphate‐binding domain may be present which binds to and stabilizes the head group of the donor phospholipid (Noland et al., 2017). Extending from the catalytic domain is a lid loop (Lu et al., 2017) that is the most variable and flexible region between the multiple crystal structures, and contains several essential residues (Gelis‐Jeanvoine et al., 2015; Lu et al., 2017; Vidal‐Ingigliardi et al., 2007). It is observed resting on the membrane and also in two increasingly raised positions that may correlate with the proposed bound states of the substrates (Wiseman and Hogbom, 2020) echoing the flexibility also seen by molecular dynamics (Lu et al., 2017; Noland et al., 2017). The flexible nature of the lid loop may control entry of substrates into the active site (Lu et al., 2017; Wiseman and Hogbom, 2020). Wiseman and Hogbom (2020) propose that movement of this loop into its upward position creates a restricted access window allowing only apolipoprotein accommodation. TH3 and TH4 extend into the periplasm forming a portal for amphiphilic substrates (Wiktor et al., 2017) and various arms create an opening to the membrane playfully described as reflecting a hungry octopus (Wiktor et al., 2017; Wiseman and Hogbom, 2020). Noland et al. (2017) describe a gating phenylalanine and proposes a mechanism whereby a flexible loop, with F82 in the open position, allows phosphatidylethanolamine to bind the lipid binding groove and moves into the active site. F82 closes and positions the *sn*‐1‐acyl chain for nucleophilic attack by C387 generating acyl‐Lnt. Then, in the open position, lyso‐PE exits the enzyme allowing the entry of the fatty acid modified cysteine of apolipoprotein via the lipid channel. However, the observed gating by F82 was not correlated with the presence or absence of substrate (Wiseman and Hogbom, 2020) and is noncritical to activity (Noland et al., 2017).

Lnt activity was first demonstrated in detergent solubilized membrane vesicles with apolipoprotein substrates obtained from globomycin‐treated cells (Gupta and Wu, 1991). The difference in temperature stability between Lsp and Lnt allowed for the accumulation of apolipoprotein substrate upon incubation at elevated temperatures. This study demonstrated the incorporation of palmitic acid from phospholipid through an amide bond in S‐diacylglyceryl‐cysteine. The initial determination of kinetic parameters of Lnt of *E. coli* was performed with an activity test based on purified Lnt, a synthetic biotinylated peptide (fibroblast‐stimulating ligand 1 or FSL‐1) and commercial phospholipids (Hillmann et al., 2011). The mobility shift of FSL‐1 upon N‐acylation by Lnt was monitored by high‐resolution gel electrophoresis (Sankaran and Wu, 1994) and detection with streptavidin. Phosphatidylethanolamine was observed as the preferred acyl donor (Jackowski and Rock, 1986) with saturated fatty acids on *sn*‐1 and unsaturated fatty acids on *sn*‐2 (Hillmann et al., 2011). This test was recently developed into a fluorescence‐based assay by using alkyne phospholipid as substrate and click‐chemistry to render the N‐acyl biotin peptide fluorescent, and could be detected in a sensitive manner on streptavidin‐coated multi‐well plates in a HTS compatible format (Nozeret et al., 2019; 2020).

## 4 REACHING THE FINAL DESTINATION

In proteobacteria, the majority of lipoproteins are located in the outer membrane (OM), either in the inner leaflet of the membrane facing the periplasm, or exposed on the cell surface (Wilson and Bernstein, 2016). The nature of the +2 residue in the lipobox, and in some bacteria residues at +3 and +4, determine whether the lipoprotein is retained in the inner membrane or translocated to the OM (Narita and Tokuda, 2007; Tokuda and Matsuyama, 2004). A designated ABC‐transporter, termed the Lol‐machinery, is involved in translocation of lipoproteins to the OM. The Lol‐machinery is generally composed of two integral membrane proteins LolC and LolE that together with ATP‐ase LolD release lipoproteins from the cytoplasmic membrane to the periplasmic chaperone LolA, which transfers the protein to the OM receptor LolB (Okuda and Tokuda, 2011) (Figure 1). LolB is not strictly conserved, suggesting that other OM receptors or alternative translocation pathways exists (Grabowicz, 2019; Liechti and Goldberg, 2012). Recent findings identified LolF as an alternative component of the ABC transporter that contains structural characteristics of both LolE and LolC and functions alongside LolD (LoVullo et al., 2015; McClain et al., 2020). Interestingly, in LolF containing bacteria, Lnt is not essential for viability, suggesting that LolF and LolD can release diacylated lipoproteins from the membrane (LoVullo et al., 2015). Lipoprotein trafficking to the OM can also occur through a LolAB‐independent mechanism in certain mutant backgrounds (Grabowicz and Silhavy, 2017). Furthermore, in *Neisseria* a designated OM and surface transport machinery exists called SLAM (surface lipoprotein assembly modulator) (Hooda and Moraes, 2018) and in spirochetal species a Lol‐independent proposed coupled “holding‐flipping” machinery locates lipoproteins to the cell surface (Zuckert, 2014).

Three independent phenotypic screens identified inhibitors of *E. coli* growth that target the Lol machinery (McLeod et al., 2015; Nayar et al., 2015; Nickerson et al., 2018). The screens used bacteria with a permeabilized OM, either through a mutation or treatment with antimicrobial peptide to allow access of the molecules, or reduced ability to efflux toxic compounds through a mutation in an RND efflux pump. Pyridine imidazole compounds were shown to interfere with LolE and LolC (McLeod et al., 2015), and a pyrazole compound inhibits the LolCDE complex (Nayar et al., 2015). These results demonstrate the importance of OM lipoproteins in cell wall biogenesis and viability. Another inhibitor, pyrrolopyrimidinedione compound (G0507), targets LolCDE and stimulates LolD ATPase activity in vitro (Nickerson et al., 2018).

Inhibitory molecules of the Lol machinery were also identified in a chemical genomics approach. Overproduction of essential proteins were identified as suppressors of inhibition of growth in the presence of small molecules (Pathania et al., 2009). Molecule MAC13243 and its degradation products bind to the hydrophobic cavity of LolA, preventing interaction with lipoproteins (Barker et al., 2013; Pathania et al., 2009). The thiourea degradation product of MAC is an A22 analog that inhibits actin homolog MreB. A22 also acts on LolA (Barker et al., 2013). Molecular dynamic simulation experiments suggest that MAC13243 and lipoproteins occupy the LolA binding site simultaneously, that conformational changes in LolA upon lipoprotein binding are restricted, and that the interaction with lipoprotein is weakened (Boags et al., 2019). The MAC13243‐LolA interaction leads to an increase in OM permeability (Muheim et al., 2017). The crucial role of the lipoprotein biosynthesis pathway is seen through these inhibitor studies of the downstream processes, and further affirms the potential antimicrobial benefits of targeting this pathway.

## 5 TARGETING THE PATHWAY

The essential nature of the lipoprotein posttranslational modification pathway in proteobacteria makes it an intriguing novel target for antimicrobial therapy. Another advantage is the accessibility of the active sites from the periplasm as molecules need not traverse the cytoplasmic membrane. To date, globomycin and myxovirescin are the only inhibitors of the lipoprotein modification pathway, both targeting Lsp, but neither are in clinical use. Clinical trials for the treatment of gingivitis by myxovirescin did shown some promise (Manor et al., 1989). Stability, effectiveness and toxicity in host cells has proven to be an obstacle for antimicrobial peptides (Chen and Lu, 2020) as is probably the case for these compounds.

Until recently, assays developed to study the pathway involved radiolabeling and gel‐shift analysis. These assays, albeit a very valuable tool, are low‐throughput, and therefore, not suitable for HTS applications. The nature of the lipoprotein modification reactions is complex. The enzymes are integral membrane proteins, and the peptide substrates and phospholipids are also components of the lipid bilayer. It is therefore not straightforward to develop assays that are simple, homogeneous, soluble, and adaptable for the screening of inhibitors. A coupled fluorescence‐based assay has been developed for Lgt that may be adapted for HTS (Sundaram et al., 2012) although no applications have been reported. Recently, an in vitro HTS Lsp assay was developed using FRET and was used to screen over 640,000 molecules for Lsp inhibition. This study yielded promising results (Kitamura et al., 2018). A fluorescence‐based click‐chemistry assay compatible for HTS has been developed for Lnt activity and is a promising tool for screening libraries of molecules (Nozeret et al., 2019; 2020). However, a drawback to target‐based in vitro screening is the potential need to chemically alter inhibitors to enable passage of the OM and to access the periplasm of proteobacteria. The identification of targets of inhibitors found in phenotypic screens requires whole genome sequencing of resistant clones. Furthermore, the active compounds described so far are only able to prevent growth of bacterial cell cultures in the presence of a permeable outer membrane. An alternative approach is the use of structure‐based drug design (Staker et al., 2015). Since the structures of the lipoprotein modification enzymes are known there is greater information available for this approach and has been reviewed in detail recently (El Arnaout and Soulimane, 2019). Ideally, these approaches should be used in parallel in the search for novel antibacterial agents.

## 6 CONCLUSIONS

Tremendous progress has been made in recent years on the structural understanding of the lipoprotein modification enzymes. Some insights have been obtained in the molecular mechanism of the reactions, in particular on phospholipid substrate specificity for Lgt and Lnt, and inhibition of Lsp by globomycin and myxovirescin. The results with globomycin suggest that the same enzyme from different bacterial species may differ in substrate specificity and efficacy of the reaction. Furthermore, it is unknown how the enzymes bind the peptide substrates and how this affects conformational changes and catalysis of the reactions. In most studies on lipoprotein modification and targeting, Braun's lipoprotein of *E. coli* is used as a model protein but it is far from being conserved among bacteria. However, interesting similarities are seen between the three enzymes, such as the arm domains or channels, which allow entry of substrate and phospholipids, and the flexibility of extended loops presumably permitting different substrates into close proximity of their active sites. To date, all studies have been conducted in isolation and there is little to no research into the functional interactions between the enzymes. The efficient nature of the system, and relative low abundance of the enzymes but high abundance of lipoproteins, hints toward a coordinated relationship to guarantee efficient lipidation of proteins as suggested in 1982 by Tokunaga (Tokunaga et al., 1982).

The development of tools to study this essential pathway has yielded the identification of inhibitors, which demonstrates progress in the race to develop or discover novel antibiotics. The combined, synergistic use of inhibitors targeting the lipoprotein modification and OM sorting pathways could be one method to increase efficacy of treatment and reduce frequency of resistance. Examples of successful combination therapy or a multi target approach have been reported (Tyers and Wright, 2019). Colistin, for example, is membrane‐permeabilizing agent, which increases drug access to the cell that has been used in combination with other antibiotics. Alongside the identification of novel antibiotics, inhibitors are a useful tool in elucidating molecular mechanisms of proteins, and in the study of complex pathways. We believe the potential for inhibiting this pathway and the recent advances in our understanding make the lipoprotein modification pathway an exciting area for future study, and may play a key role in the fight against antimicrobial resistant pathogens.

## CONFLICT OF INTEREST

The authors have no conflict of interest to declare.
